# DNA extraction from primary liquid blood cultures for bloodstream infection diagnosis using whole genome sequencing

**DOI:** 10.1099/jmm.0.000664

**Published:** 2018-01-10

**Authors:** Luke W. Anson, Kevin Chau, Nicholas Sanderson, Sarah Hoosdally, Phelim Bradley, Zamin Iqbal, Hang Phan, Dona Foster, Sarah Oakley, Marcus Morgan, Tim E. A. Peto, Modernizing Medical Microbiology Informatics Group (MMMIG), Derrick W. Crook, Louise J. Pankhurst

**Affiliations:** ^1^​ Nuffield Department of Clinical Medicine, University of Oxford, John Radcliffe Hospital, Oxford, OX3 9DU, UK; ^2^​ Wellcome Trust Centre for Human Genetics, University of Oxford, Oxford OX3 7BN, UK; ^3^​ NIHR Health Protection Unit in Healthcare Associated Infections and Antimicrobial Resistance at University of Oxford in partnership with Public Health England, Oxford, UK; ^4^​ Microbiology Laboratory, John Radcliffe Hospital, Oxford University Hospitals NHS Trust, Oxford, OX3 9DU, UK; ^5^​ National Institute for Health Research (NIHR) Oxford Biomedical Research Centre, John Radcliffe Hospital, Oxford, OX3 9DU, UK; ^6^​ Public Health England, Wellington House, 133-155 Waterloo Rd, Lambeth, London SE1 8UG, UK; ^†^​ Present address: Genomic Research Laboratory, Division of Infectious Diseases, University of Geneva Hospitals, Rue Gabrielle-Perret-Gentil, 4, CH-1211 Geneva 14, Switzerland.

**Keywords:** bloodstream infection, sepsis, bacteraemia, whole genome sequencing

## Abstract

**Purpose:**

Speed of bloodstream infection diagnosis is vital to reduce morbidity and mortality. Whole genome sequencing (WGS) performed directly from liquid blood culture could provide single-assay species and antibiotic susceptibility prediction; however, high inhibitor and human cell/DNA concentrations limit pathogen recovery. We develop a method for the preparation of bacterial DNA for WGS-based diagnostics direct from liquid blood culture.

**Methodology:**

We evaluate three commercial DNA extraction kits: BiOstic Bacteraemia, Amplex Hyplex and MolYsis Plus. Differential centrifugation, filtration, selective lysis and solid-phase reversible immobilization bead clean-up are tested to improve human cells/DNA and inhibitor removal. Using WGS (Illumina/MinION), we assess human DNA removal, pathogen recovery, and predict species and antibiotic susceptibility inpositive blood cultures of 44 Gram-negative and 54 *Staphylococcus* species.

**Results/Key findings:**

BiOstic kit extractions yield the greatest mean DNA concentration, 94–301 ng µl^−1^, versus 0–2.5 ng µl^−1^ using Amplex and MolYsis kits. However, we note higher levels of inhibition (260/280 ratio 0.9–2.1) and human DNA (0.0–4.4×10^6^ copies) in BiOstic extracts. Differential centrifugation (2000 ***g***, 1 min) prior to BiOstic extraction reduces human DNA by 63–89 % with selective lysis minimizing by a further 62 %. Post-extraction bead clean-up lowers inhibition. Overall, 67 % of sequenced samples (Illumina MiSeq) contain <10 % human DNA, with >93 % concordance between WGS-based species and susceptibility predictions and clinical diagnosis. If >60 % of sequencing reads are human (7/98 samples) susceptibility prediction becomes compromised. Novel MinION-based WGS (*n*=9) currently gives rapid species identification but not susceptibility prediction.

**Conclusion:**

Our method for DNA preparation allows WGS-based diagnosis direct from blood culture bottles, providing species and antibiotic susceptibility prediction in a single assay.

## Introduction

Bacterial bloodstream infections (BSIs) are a major cause of morbidity and mortality [1, 2]. In England, Public Health England (PHE) report the 2015/16 30-day mortality rate of BSI of major pathogens (*Escherichia coli,* methicillin-sensitive *Staphylococcus aureus* and methicillin-resistant *S. aureus*) as 14.7, 19.7 and 28.1% respectively, with *E. coli* mortality highest overall (5738 deaths) [3]. Early appropriate antimicrobial therapy is crucial to reduction of BSI-related mortality and morbidity, length of stay and healthcare costs [4–10]. However, review of empirical BSI therapy follows phenotypic identification and antimicrobial susceptibility testing (AST) of bacterial species, a time-consuming process that delays targeted therapy. Given empirical therapy may be inappropriate in up to 40 % of cases [5], and the continued growth in the prevalence of antimicrobial resistant bacteria [11], an earlier informed and targeted therapy for BSI is becoming increasingly important [4–10].

Methods for rapid, automated, organism identification and phenotypic testing, such as matrix-assisted laser desorption/ionization mass spectrometry (MALDI-TOF MS; e.g. Bruker, USA) and automated microbroth dilution (e.g. BD Phoenix; BD, USA) respectively, reduce turnaround time and demonstrate the positive benefits associated with earlier appropriate therapy [12]. Despite these advances, reliance on pure microbial culture continues. Depending on the micro-organism, conventional laboratory workflows can therefore vary from 24 h to several weeks to complete [5–13].

Species identification and AST methods directly from primary blood culture have the potential to reduce turnaround times, and show benefits in clinical care [4–10]. For example, MALDI-TOF methods have been adapted for this purpose, with varying degrees of accuracy [14, 15]. However, MALDI-TOF cannot provide full drug susceptibility information from primary culture [4, 6–8]. Microarray and PCR-based molecular tests target panels of species-specific and drug-resistance markers in primary culture [8, 9]. Although molecular methods report rapid and accurate diagnosis [8, 9], panel sizes are limited and none encompass the full diversity of BSI-causing bacteria and drug-resistance markers (for examples see [16–21]). Sequence divergence in the primer region may affect sensitivity of these assays whereas the impact of DNA contamination, from both human and other bacterial cells, affects the specificity. Thus, molecular approaches continue to present a challenge [22, 23].

Whole genome sequencing (WGS) offers a solution to the limitations of PCR-based methods, providing species identification that is un-restricted by a target panel, with the advantages of antimicrobial susceptibility prediction, lineage and information regarding relatedness to other isolates using the same data. Retrospective investigations for pure cultures of *E. coli*, *Klebsiella pneumoniae* and *S. aureus* demonstrate that WGS diagnostic accuracy is comparable to routine phenotyping methods [24, 25]. WGS directly from primary blood culture could reduce turnaround times to a clinically applicable time-frame. However, this is more challenging, reliant on the removal of a diverse range of inhibitors present in the liquid media [7, 26], and depletion of human cells and/or DNA to allow recovery of sufficient good-quality pathogen DNA. Initial bacterial density in BSI falls between 1–100 c.f.u. ml^−1^ [27] and even post-primary culture recovery of bacterial DNA for successful WGS is complicated by the presence of large amounts of human DNA.

We report a method to deplete human cells/DNA and isolate bacterial DNA of sufficient quality and quantity for WGS direct from primary liquid blood culture. We demonstrate the ability of the method to provide species identification using short-read Illumina sequencing and the emerging long-read Nanopore MinION system, as well as antimicrobial-resistance prediction using Illumina sequencing data.

## Methods

### Sample collection and processing

Positive blood culture specimens identified as containing either Gram-negative organisms or *Staphylococcus* sp. by routine clinical Gram-stain were retrieved from the Oxford University Hospitals National Health Service (UK) (NHS) Foundation Trust microbiology laboratory. Blood culture bottles collected included the BD BACTEC Aerobic Plus, Peds Plus, and Lytic Anaerobic (BD, USA). The former two contain resin, and the latter lytic agents for the release of intracellular pathogens. Positive samples from the previous 24 h were retrieved by 10am each working day. Samples were stored at 37 °C between positivity and collection, 5 ml of culture was immediately removed and retained for processing.

### DNA extraction and pre-steps

Three DNA extraction and purification kits were tested: MolYsis Plus kit (Molzym, Germany), BiOstic Bacteraemia kit (MoBio, Qiagen, USA) and Amplex Hyplex Quickprep (Amplex Biosystems, Germany) following the manufacturers’ protocol. Chosen kits were readily available in the UK and designed to remove inhibitors to molecular testing. DNA extracted using BiOstic Bacteraemia kit was analysed with and without subsequent DNA purification using AMPure XP solid-phase reversible immobilization (SPRI) beads following the manufacturer’s protocol (Beckman Coulter, UK). BiOstic kit extractions were also performed with pre-treatment steps for the depletion of human DNA. These steps were differential centrifugation of primary culture, selective lysis of the primary culture pellet, filtration of primary culture, and a combination of the methods (fully detailed in Fig. S1, available in the online version of this article). DNA was quantified and qualified using the NanoDrop 1000 or Qubit 2.0 fluorometer (double-stranded DNA high sensitivity/broad range kits as required; Thermo Fisher Scientific, USA).

### PCR

Quantitative PCR using the probes and primers described in Table S1 was used to quantify *S. aureus*, *E. coli* or 16S rRNA, as well as human GAPDH or β-actin DNA (Fig. S1). *S. aureus, E. coli* and human GAPDH primers and probes were used at a final concentration of 0.32 µM, 16S primers and a probe were used at 0.1 µM, human β-actin primers were used at 0.5 µM and the probe at 0.2 µM. All reactions used 1X Brilliant Multiplex qPCR Master Mix (Agilent, USA) with 2 µl DNA and sufficient molecular grade water to bring the reaction volume to 25 µl. Amplification was performed using the MxPro 3005P (Agilent, USA) under the following conditions: 95 °C for 10 min, 40 cycles of 95 °C for 15 s and 60 °C for 1 min. Extractions showing evidence of inhibition were re-amplified using 2 µl of 1 : 10 and 1 : 100-fold diluted DNA, and following SPRI bead clean-up.

### Illumina MiSeq whole genome sequencing

The finalized protocol (Fig. S1) was used to prepare samples for WGS (finalized protocol, Fig. S1). Bacteria and other debris were pelleted via differential centrifugation of primary culture (1000 ***g***, 1 min). The pellet was retained, re-suspended in 1 ml molecular grade water and incubated at room temperature for 5 min to selectively lyse human cells. The suspension was re-pelleted (17 000 ***g***, 3 min) and the pellet taken forwards to DNA extraction, or re-suspended in 1 ml nutrient broth with 10 % glycerol and stored at −20 °C until extraction. DNA extraction was performed using the BiOstic kit followed by SPRI bead clean-up. Sequencing libraries were prepared using the Nextera XT kit (Illumina, USA) following the manufacturer’s protocol with manual library normalization. Sequencing was performed using MiSeq v2 2×150 base pair (bp) paired-end read cartridges and MiSeq v3 2×75 or 2×300 bp paired-end read cartridges.

### MinION whole genome sequencing

As DNA input requirements for MinION sequencing are higher than for MiSeq, 10 ml blood culture was processed per sample. *S. aureus* and *E. coli* DNA extracts (finalized protocol, Fig. S1) were prepared for MinION sequencing using the Rapid Sequencing Kit (SQK-RAD002; RSE_9018_v2_redD_21Nov2016) following the manufacturer’s protocol. Sequencing was performed using R9.4 flow cells for 48 h and sequencing reads live basecalled in MinKNOW version 1.2–1.3 (ONT, UK).

### Sequence analysis

MiSeq sequencing data was processed via an in-house pipeline. Reads were classified with the metagenomic classifier, Kraken (database built from bacterial, viral and human genomes present in National Center for Biotechnology Information refseq on 14 January 2015; v0.10.6-unreleased) [28], and human reads removed. Remaining reads were mapped (stampy v1.0.23) to a reference genome chosen according to the top species hits from Kraken (for *S. aureus* GenBank BX571856.1 and for *E. coli* GenBank AE014075.1). For *Staphylococcus* sp. specimens, the species was also predicted via the publically available Mykrobe Predictor [29]. Mykrobe also predicts antibiotic susceptibility for *S. aureus* (Mykrobe v0.3.6–0-g9d196c7), while antibiotic susceptibility for *E. coli* and *Klebsiella* sp. was predicted using resistType (https://github.com/hangphan/resistType), an in-house algorithm using a previously published catalogue of resistance conferring mutations/genes [25]. We determined sequencing quality through assessment of overall genome coverage and depth of coverage from both Mykrobe and mapping analysis, as well as the total number of reads available.

Predictions of genotype (species and antimicrobial resistance from Mykrobe for *Staphylococcus* sp.; species from Kraken and antimicrobial resistance from resistType for *E. coli* and *Klebsiella* sp.) were compared to anonymized clinical diagnosis generated using pure culture isolates [species from Bruker microflex MALDI-TOF MS (Bruker, USA), antimicrobial resistance from BD Phoenix microbroth dilution (BD, USA)]. In all cases the clinical diagnosis was taken as the gold standard comparator method. The sensitivity (clinical positives/clinical positives+WGS false negatives) and specificity (clinical negatives/clinical negatives+WGS false positives) of WGS-based diagnosis was calculated. When analysing concordance between Kraken data and clinical species identification, we disregarded organisms with <1 % of available reads assigned to them.

For MinION-generated reads fastq and timing data was extracted in real time and iteratively updated using fast5Watcher.py (https://github.com/nick297/fast5_scripts; commit vb88e14a). We conducted metagenomic classification using Kraken with no filtering threshold. We predicted antibiotic susceptibility via Mykrobe for samples identified as *S. aureus* (v0.4.3–0-gd6c8714), and for *E. coli* and *Klebsiella* sp.-resistance predictors were identified by blast against the published mutation/gene catalogue [25]. Mykrobe provided data quality parameters including depth of species and resistance mechanisms for *S. aureus*. The number of bases recovered, read number and length statistics, and accuracy, were calculated for all runs using nanoStats.py (https://github.com/nick297/fast5_scripts; commit vb88e14a).

## Results

### DNA extraction optimization

Measurement of DNA yields from 23 positive blood cultures (*E. coli, n*=11, *S. aureus, n*=12) from 17 individuals (aerobic and anaerobic blood culture processed from 6/17 individuals) followed extraction using the three commercially available kits. According to manufacturers, all kits remove inhibitors and deplete human cells.

Initial yields from six *E. coli* and six *S*. *aureus* positive samples demonstrated that the BiOstic kit provides the most DNA (Qubit fluorometer), with mean values up to 430x greater than MolYsis or Amplex (Table 1). All BiOstic extracts contained detectable DNA, while in 2/6 MolYsis and 5/6 Amplex no *S. aureus* DNA was detected. On this basis, Amplex was disregarded as a suitable method to extract DNA from blood cultures for WGS purposes.

**Table 1.** DNA yield for 12 samples extracted using Amplex Hyplex Quickprep, BiOstic Bacteraemia, and MolYsis Plus kits Mean, sd and inter-quartile range (IQR) values were measured using the Qubit double-stranded DNA high sensitivity or broad range quantification kit shown (kits used as required). 0=DNA below detection limits of high sensitivity quantification kit.

**Measurement**	**Sample numbers**	**Extraction kit**	***E. coli***			***S. aureus***		
			**Mean**	**sd**	**IQR**	**Mean**	**sd**	**IQR**
Qubit (ng µl^−1^)	6x *E. coli*, 6x *S. aureus*	Amplex	0.7	0.3	0.6–1.0	0.0	0.1	0.0–0.0
		BiOstic	300.5	78.8	275.0–335.0	94.0	90.3	4.8–118.0
		MolYsis	2.5	4.7	0.5–0.9	0.2	0.1	0.0–0.2

qPCR assessment of the 12 initial extracts was performed using *S. aureus, E. coli* and human GAPDH targets (Table S1). MolYsis extracts yielded 10^3^–10^5^ copies [inter-quartile range (IQR)] for *E. coli* and *S. aureus*; alongside 0–10^2^ human copies (IQR; Table 2). The six BiOstic extracts initially failed to amplify, but amplification of diluted input DNA with and without SPRI bead clean-up (*n*=4) yielded 10^3^–10^7^ copies of *S. aureus* and *E. coli*; alongside 0–10^6^ human copies (IQR; Table 2). This suggests amplification may be inhibited by contaminants carried over during the extraction process or from excessive DNA.

**Table 2.** qPCR copy number data samples extracted using MolYsis Plus and BiOstic Bacteraemia kits; human DNA measured using GAPDH qPCR target (Table S1). Mean, sd and inter-quartile range (IQR) values are shown. 0=DNA undetectable. n/a=data not available as only two samples tested.

	**Extraction kit**	**Bacterial copy number**			**Human copy number**			
		**Mean**	**sd**	**IQR**	**Mean**	**sd**	**IQR**
***S. aureus***	MolYsis (*n*=6)	9.3×10^4^	7.7×10^4^	5.7×10^3^–1.6×10^5^	6.0×10^2^	9.6×10^2^	0.0–5.4×10^2^
	BiOstic (*n*=6)	4.0×10^7^	4.4×10^7^	0.0–8.4×10^7^	5.0×10^6^	8.7×10^6^	0.0–4.4×10^6^
	BiOstic 1 : 10 (*n*=6)	7.1×10^6^	5.7×10^6^	1.5×10^5^–1.2×10^7^	7.3×10^5^	1.2×10^6^	2.0×10^3^–5.8×10^5^
	BiOstic 1 : 100 (*n*=6)	8.1×10^5^	6.3×10^5^	2.4×10^4^–1.3×10^6^	6.9×10^4^	1.2×10^5^	3.9×10^2^–5.3×10^4^
	BiOstic+SPRI 1 : 10 (*n*=2)	3.3×10^6^	4.6×10^6^	n/a	6.2×10^5^	8.7×10^5^	n/a
	BiOstic+SPRI 1 : 100 (*n*=2)	3.4×10^5^	4.6×10^5^	n/a	6.9×10^4^	9.6×10^4^	n/a
***E. coli***	MolYsis (*n*=6)	5.8×10^5^	7.7×10^5^	1.7×10^5^–4.5×10^5^	4.0×10^2^	5.6×10^2^	0.0–4.0×10^2^
	BiOstic (*n*=6)	0.0	0.0	0.0–0.0	0.0	0.0	0.0–0.0
	BiOstic 1 : 10 (*n*=6)	1.4×10^7^	8.5×10^6^	6.5×10^6^–1.9×10^7^	2.2×10^5^	3.3×10^5^	1.2×10^4^–3.7×10^5^
	BiOstic 1 : 100 (*n*=6)	2.1×10^6^	9.9×10^5^	2.0×10^6^–2.5×10^6^	3.0×10^4^	5.1×10^4^	1.9×10^3^–3.5×10^4^
	BiOstic+SPRI 1 : 10 (*n*=2)	1.9×10^7^	1.1×10^7^	n/a	2.1×10^3^	2.5×10^3^	n/a
	BiOstic+SPRI 1 : 100 (*n*=2)	1.8×10^6^	9.1×10^5^	n/a	4.1×10^2^	5.8×10^2^	n/a

Additional contamination analysis was performed using the NanoDrop on fresh MolYsis extractions (*S. aureus n*=6; *E. coli n*=5; Table S2), and BiOstic extractions with and without SPRI clean-up (*S. aureus n*=3; *E. coli n*=3; Table S2). All initial extracts had low 260/230 ratios (ranges: *S. aureus* 0.1–2.2, *E.coli* 0.1–2.3) with low 260/280 ratios also seen in BiOstic extracts (IQR *S. aureus* 0.2–2.2, *E.coli* 0.9–2.1; Table S2); indicating carry-over of contaminants from the extraction process. DNA yields in BiOstic extracts were 30x higher than MolYsis, allowing SPRI clean-up to improve DNA purity. Clean-up reduced mean DNA yield to 39.3 ng µl^−1^ for *E. coli* and 11.5 ng µl^−1^ for *S. aureus*, but increased purity, indicated by higher 260/280 (IQR *S. aureus* 1.4–2.2, *E.coli* 1.9–2.2; Table S2) and 260/230 ratios (IQR *S. aureus* 1.9–2.7, *E.coli* 2.2–2.3; Table S2).

Overall, yields in *E. coli* samples exceeded *S. aureus* from both extraction kits, possibly due to incomplete lysis of *S. aureus* without a specific lysis enzyme (e.g. lysostaphin). The MolYsis kit was more successful at human cell/DNA depletion, shown by the lower ratio of human to bacterial DNA as compared to BiOstic (Table 2). However, the higher bacterial DNA concentrations and the success of SPRI clean-up of BiOstic extractions indicates the validity of this method for further testing to enhance human cell depletion.

### Depletion of human cells/DNA optimization

All human cell/DNA depletion experiments were assessed using qPCR (Fig. S1, Tables S1 and S3). Differential centrifugation was performed for two fresh samples to determine what speed and time effectively depleted human copy number (Fig. S1; pre-step a) from initial values of 248 in sample 1 and 56 in sample 2. In sample 1, 2000***g*** centrifugation, for both 30 s and 1 min demonstrated the greatest reduction in human copy number (63–77 %; Table S3) whereas in sample 2 centrifugation at 1000 ***g*** for 30 s and 1 min reduced copy number by >90 %. Variation in relative bacterial load was minimal; other than in 3000 ***g*** for 1 min centrifugation which depleted bacterial cells (Table S3).

Inclusion of an additional distilled water wash (Fig. S1 pre-step b) resulted in an extra 62 % (mean; range 37–80 %; *n*=7) depletion of human cells (data not shown).

Fresh *S. aureus* positive specimens were collected to assess the efficacy of a filtration step (Fig. S2; pre-step c). However, filtration resulted in variability in *S. aureus* DNA yield (IQR 6.7×10^4^–1.0×10^7^ versus 2.9×10^6^–4.0×10^6^; *n*=6) (qPCR; Table S1). Therefore, the filtration method was not taken further in this study.

Although 1000 ***g*** centrifugation resulted in the largest single reduction in human copy number seen across both samples, centrifugation at 2000 ***g*** reduced human DNA more effectively where burden was higher and was consequently incorporated into the final protocol alongside a distilled water wash (Table S3 and finalized protocol in Fig. S1).

### Illumina MiSeq whole genome sequencing

MiSeq-based WGS on newly collected positive blood culture samples (*Staphylococcus* sp. 54 specimens, 51 individuals; Gram-negative bacteria 44 specimens, 42 individuals) measured the effectiveness of the final extraction process (Fig. S1; finalized protocol). Read numbers ranged from 1.5 to 5.2 million per sample (v2 cartridges: 1.5–3.73 million; v3 cartridges: 3.1–5.2 million). All repeat specimens from the same individual were identical in terms of species and drug susceptibility prediction.

**Fig. 1.**
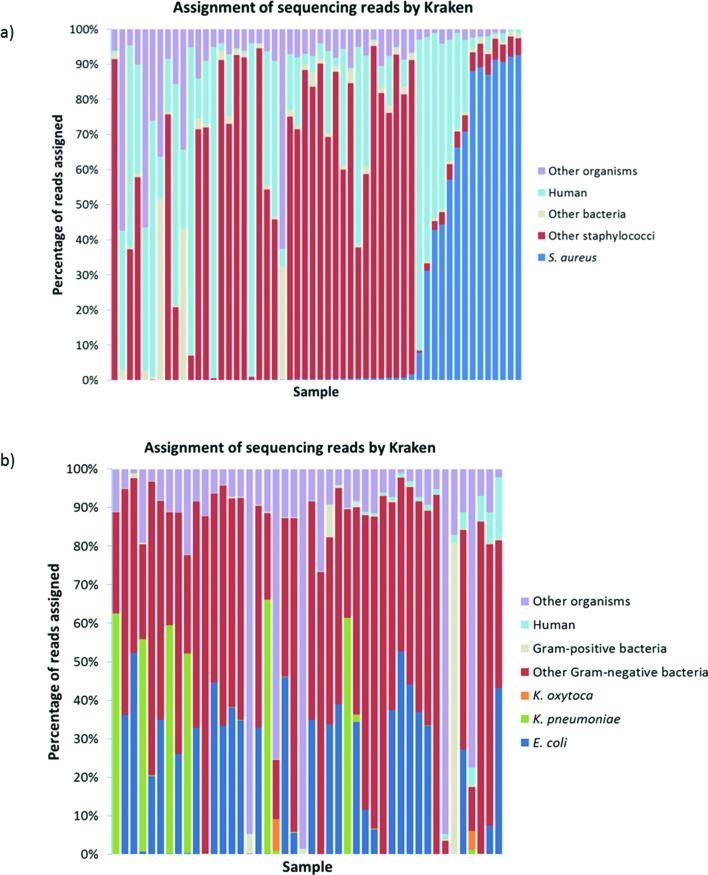
Assignment of reads by Kraken metagenomics analysis. (a) Gram-positive blood cultures (*n*=54). Reads are categorized as follows: *S. aureus*, other staphylococci (identification to genus level only, or non-*S. aureus*), other bacteria, human and other organisms (e.g. viruses). (b) Gram-negative blood cultures (*n*=44). Reads are categorized as follows: *E. coli*, *K. pneumoniae, K. oxytoca,* other Gram-negative bacteria, Gram-positive bacteria, human and other organisms (e.g. viruses).

Mykrobe [29] identified *S. aureus* with 100 % sensitivity and 98 % specificity in comparison to routine clinical identification [*S. aureus* identified by WGS in 14/14 routine clinical *S. aureus* positives+1 *S. aureus* identified by WGS only (‘false-positive’); Table 3]. Mykrobe achieved >99 % reference genome coverage across all 14 *S. aureus* samples, with 13–152x depth; conversely the single additional *S. aureus* found had 24 % reference genome coverage and 1x depth. This may represent a false-positive call by Mykrobe, or the presence of *S. aureus* at a low concentration. Across all 54 samples, Kraken [28] analysis assigned 0.01–95 % (mean 24.5 %, IQR 2.3–39.5 %) of sequencing reads to human DNA (Fig. 1).

**Table 3.** Summary of target species (*Staphylococci* sp., *E. coli* and *Klebsiella* sp.) identified by clinical diagnosis (MALDI-TOF) and WGS; full species breakdown provided in Table S4

**Species identified**	**No. identified by MALDI-TOF**	**No. identified by WGS**
Gram-positive blood cultures (*n*=54)		
Coagulase negative *Staphylococci*	37	39
*Staphylococcus aureus*	14	15
Other	3	0
Gram-negative blood cultures (*n*=44)		
*Escherichia coli*	20	24
*Klebsiella pneumoniae*	4	5
*Klebsiella oxytoca*	1	0
*Klebsiella pneumoniae+Klebsiella oxytoca*	0	1
*Escherichia coli+Klebsiella* sp.	3	1
*Escherichia coli*+other (non-*Klebsiella* sp.)	5	2
*Klebsiella* sp.+other (non-*Escherichia coli*)	2	2
Other (non-*Escherichia coli* and non-*Klebsiella* sp.)	9	9

*E. coli* and *Klebsiella* sp. identification was 100 and 90 % sensitive (*E. coli* identified by WGS in all 27 clinical positives; *Klebsiella* sp. identified by WGS in 8/9 clinical positives), and 100 and 97 % specific respectively (0 additional *E. coli* specimens detected by WGS; 1 additional *Klebsiella* sp. specimen detected by WGS; Tables 3 and S4). Overall, Kraken assigned the same organism as routine clinical diagnosis in 93 % (41/44) of specimens (Table S4). Three non-*E. coli* and non-*Klebsiella* sp. specimens were discrepant (MALDI-TOF *Aeromonas* sp., *Acinetobacter lwoffi, and Brevibacillus* sp.; Kraken *Enterobacter aerogenes*, *Acinetobacter baumannii*, and unclassifiable). Polymicrobial infections were identified by clinical diagnosis in 12/44 cases (including two non-*E. coli* and non-*Klebsiella* sp. specimens); Kraken classified all species in 2/12 of these cases, while in 3/12 cases the un-identified organism(s) were found in an additional, un-sequenced, blood culture bottle (Table S4). For the remaining 7/12 cases, the co-infecting organisms were found at <0.5 % of the total read number (3/7), or were undetectable by Kraken (4/7). Kraken identified additional organisms, not seen in clinical diagnosis, in 4/44 of cases (although at 1–2 % of the total read number). The percentage of human DNA reads across all samples ranged from 0–15.7 % (mean 1.2 %, IQR 0.04–1.1 %; Fig. 1).

**Fig. 2.**
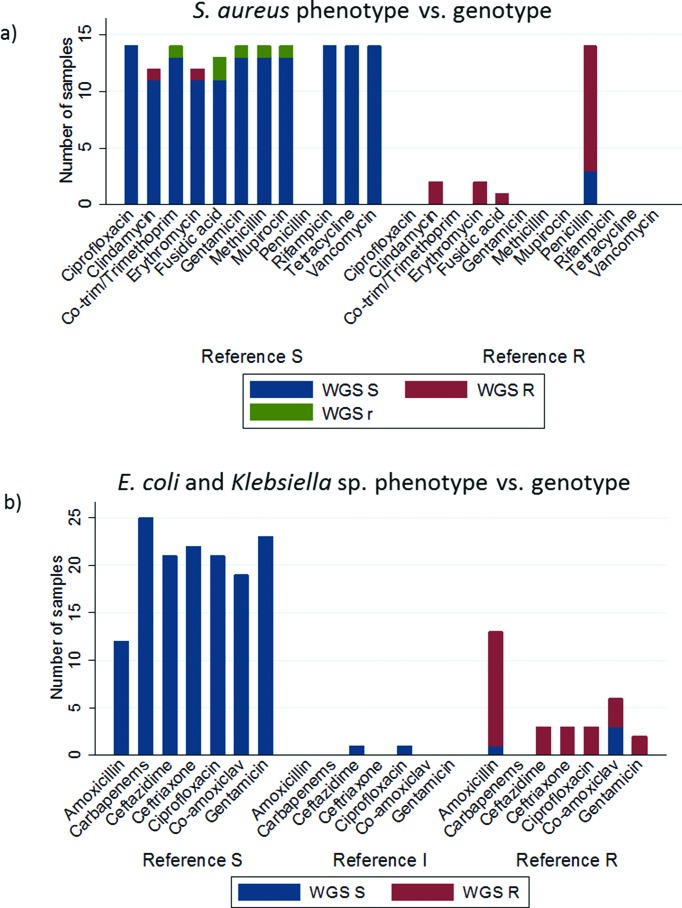
WGS predicted drug resistance as compared to phenotype. Reference S=susceptible phenotype, reference I=intermediate phenotype, reference R=resistant phenotype. WGS S (blue)=susceptible genotype, WGS R (red)=resistant genotype, WGS r (green)=low frequency resistance conferring allele found. (a) *S. aureus* phenotype determined via Phoenix. Co-trim/trimethoprim comparison based on trimethoprim-sulfamethoxazole phenotype and trimethoprim genotype prediction. Genotype predicted by Mykrobe (b) *E. coli* and *Klebsiella* sp. phenotype determined via Phoenix. Genotype predicted via resistType.

Mykrobe provided drug susceptibility predictions across 12 antibiotics (Fig. 2) for 14 *S. aureus* samples [29]. Concordance was 95 % (160/168) between Mykrobe prediction and phenotype. Of the discordant predictions, 88 % (7/8) were in a single specimen; 4/7 of these discordant predictions were low-frequency within-sample alleles (labelled ‘r’ by Mykrobe, Fig. 2). Amongst the 14 *S. aureus* isolates, depth of coverage was lowest, and human DNA contamination highest in the two specimens with discordant drug susceptibility predictions; 13–27× depth and 62–87 % human DNA content in comparison with 57–152× depth and 27–51 % human DNA content in concordant specimens.

Drug susceptibility predictions for clinically monomicrobial *E. coli* or *Klebsiella* sp. BSI (25 isolates) showed 97 % (169/175) concordance using resistType. 2/6 discordant predictions were for specimens with intermediate MIC by standard laboratory methods (ciprofloxacin MIC 1 mg ml^−1^; ceftazidime MIC 2 mg ml^−1^; WGS sensitive for both). 3/6 discordant predictions were for co-amoxiclav (genotype S and phenotype R). No relevant genetic-resistance mechanisms were seen for both the co-amoxiclav and intermediate susceptibility discordants (using the catalogue described by Stoesser *et al*. [25]). The remaining discordant prediction (amoxicillin in a *K. oxytoca* isolate) was due to BD Phoenix algorithms automatically assigning a resistant phenotype with amoxicillin MIC elevated but below breakpoint (4 mg ml^−1^; 8 mg ml^−1^ breakpoint). No genetic resistance was reported by resistType.

### Rapid sequencing with MinION

Rapid sequencing on nine blood culture positive samples (*E. coli* or *S. aureus)* using Nanopore MinION demonstrated three sequencing run failures, with 6–29 reads obtained, and six successful runs, >4000 reads (Table 4). In successful runs median 69.3×10^6^ (IQR 11.7×10^6–^3.0×10^7^) bases were recovered with 88.4–89.7 % read accuracy.

**Table 4.** MinION read statistics as generated by nanoStats.py (https://github.com/nick297/fast5_scripts; commit vb88e14a)

**Sample**	**Organism**	**Total bases**	**No. of reads**	**Average read length (bp)**	**Longest read (bp)**	**Median read length (bp)**
1	*E. coli*	11 664 010	4 762	2449	11 296	2009
2	*E. coli*	108 669 599	48 592	2236	22 752	1881
3	*S. aureus*	473 680 151	221 196	2142	25 178	1690
4	*S. aureus*	303 247 664	116 769	2597	27 285	2086
5	*E. coli*	9 902 327	4 138	2393	17 368	2020
6	Fail	58 924	29	2032	6 979	1483
7	Fail	12 539	6	2090	3 286	1810
8	Fail	29 926	21	1425	4 149	1027
9	*S. aureus*	29 940 810	12 035	2488	23 878	1884

**Fig. 3.**
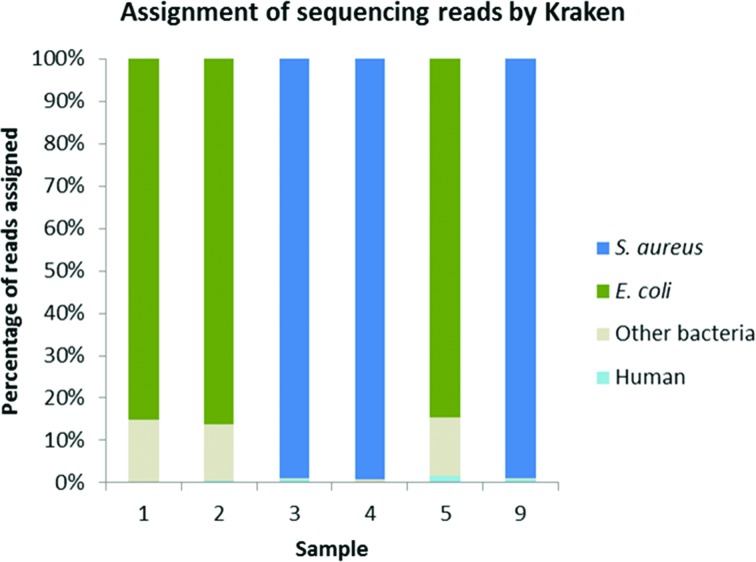
Percentage of total MinION reads assigned to *S. aureus*, *E. coli*, other bacteria, or human genomes by Kraken for samples 1, 2, 3, 4, 5 and 9. Insufficient reads for data analysis seen in samples 6, 7 and 8 (Table 4).

In successful sequencing runs over 85 % of the proportion of reads were the infecting organism (Fig. 3). Real-time analysis allowed identification of infecting species within 10 min of sequencing commencing (Fig. S2), even where yield was variable. Approximately 80 % of total yield was obtained in the first 11 h of sequencing.

Depth of coverage was 4–33x for *S. aureus* and 12–210x for *E. coli* (Table 4). Drug susceptibility prediction was 97 % concordant in *S. aureus* (samples 3, 4, 9 in Table 4; data not shown) with one specimen fully sensitive, one penicillin resistant, and one penicillin and fusidic acid resistant. Mykrobe predicted one sample, phenotypically susceptible to trimethoprim-sulfamethoxazole, to be trimethoprim resistant. Susceptibility predictions for *E. coli* were 86 % concordant (samples 1, 2, 5 in Table 4; data not shown). One isolate was fully sensitive and one concordant for amoxicillin and co-amoxiclav resistance (TEM-30 identified). In the final sample, SHV was detected, leading to a concordant prediction of amoxicillin resistance. However, the SHV variant could not be differentiated, preventing genotypic prediction for ceftriaxone, ceftazidime and co-amoxiclav.

## Discussion

We demonstrate a human cell/DNA depletion and bacterial extraction method to allow WGS directly from aerobic and anaerobic BACTEC blood culture bottles. The method includes differential centrifugation to remove intact human cells, and a distilled water wash to lyse remaining human cells. Removal of free human DNA occurs following pelleting of bacterial cells. Following these steps, we extract bacterial DNA with a commercial kit (BiOstic Bacteraemia) and clean with SPRI beads prior to sequencing; effectively removing inhibitors common to blood culture media, including sodium polyanetholsulfonate [26]. The results indicate no inhibition of WGS library preparation using this protocol. The method does not require specialist equipment or reagents, so is cost efficient and straightforward to implement in a range of settings.

Avoiding the use of specialist reagents allows the method to be used for most bacteria, exploiting the non-specificity of WGS to allow diagnosis to encompass the 20–25 pathogens causing most BSI, as well as rarer pathogens and known resistance conferring genes/mutations and virulence genes [6]. The use of specific lysis reagents, such as lysostaphin, may improve lysis of some target organisms (for example, *Staphylococcus* sp.) while kits such as the MolYsis Basic5 kit (Molzym, Germany) may reduce human DNA further. The effect of our and other potential methods on DNA concentrations for different bacterial species, including intracellular pathogens, should be explored in future investigations.

The complexity of WGS laboratory protocols and bioinformatics analysis is often viewed as an impediment to implementation in clinical settings [29, 30]. However, kit-based sequencing preparation methods (such as Nextera XT), paired with the Illumina MiSeq and bioinformatics tools designed for clinical usage (such as Mykrobe Predictor) have already enabled the roll-out of WGS-based infectious disease diagnosis for organisms such as *Mycobacterium tuberculosis* [31]. For Gram-negative bacteria a WGS-resistance prediction tool designed for clinical usage has not yet been made widely available, leading to the use of Kraken and an in-house tool (resistType) in this study. However, as an area of rapid development, we anticipate that a suitable tool will be available in the near-future [32–34].

Using our method, species identification and drug susceptibility prediction could be performed with >93 % concordance to clinical diagnostic results. We note discordance with low sequencing quality, co-infections, and high human DNA content, which reduce the number of reads available for susceptibility prediction and increases the likelihood of low-confidence predictions. For unknown reasons, the number of Gram-negative BSI identified as co-infections was high during this study at 27 % (12/44) versus reported rates of 6–12 % [19]. For 3/12 of these, phenotypic investigations identify the co-infecting organism in the un-sequenced bottle. Information regarding the distribution of species in individual culture bottles is unavailable for the remainder. Performing WGS with both aerobic/anaerobic blood culture bottles, or a mixture of the two, would avoid missing additional pathogens in the future. Although WGS may identify co-infections present in the same culture bottle more readily than MALDI-TOF MS [4], further investigations are required to confirm this potential and determine limits of detection.

WGS reports three very major errors (phenotype resistant, genotype sensitive) for co-amoxiclav resistance in Gram-negative bacteria. However, 15 % discordance between co-amoxiclav MICs generated by automated microbroth dilution and gradient diffusion has previously been observed [25]; while repetition of automated microbroth dilution generates differing susceptibility predictions in 5 % of samples [25]. There remains a clear requirement for further investigations to explore the genotype–phenotype relationship for co-amoxiclav.

The timeliness of appropriate antimicrobial therapy is crucial to the reduction of BSI-related morbidity and mortality. To this end, an 8 h turnaround from positive blood culture to full diagnosis is targeted; although the World Health Organisation and Centres for Disease Control aim for a 2 h turnaround time [5]. WGS with Illumina MiSeq currently takes 23–59 h (in this study approximately: 3 h DNA extraction; 4 h sequencing library preparation; 16–52 h MiSeq-based sequencing; Mykrobe data processing <2 min per sample). At 23 h potential turnaround time, WGS is equivalent to current culture-based methods (~24 h) and potentially offers more information. However, for MiSeq-based WGS sample batching would be required to reduce overall costs, increasing turnaround times according to local BSI specimen throughput.

An alternative approach is to explore sequencing directly from blood or plasma [35]. Sequencing circulating cell-free DNA from plasma permits species and limited antimicrobial-resistance diagnosis based on <1× coverage depth [35]. Although by-passing culture steps provides rapid turnaround, this method does not yet provide robust data for full resistance prediction.

MinION-based WGS with computational support can also reduce turnaround times; predicting species within 4 h of culture positivity (in this study: 3 h DNA extraction; 1 h for sequencing library preparation and species identification) and subsequently generating drug-resistance predictions [36]. The MinION is random access (reducing the requirement for sample batching) and permits real-time sequencing data analysis; minimizing these time-delays. This turnaround time begins to rival MALDI-TOF, even with rapid subculture [7, 10], and microarray approaches where <4 h turnaround times are reported [37]; with the advantage of being un-restricted by a target panel and capable of generating resistance predictions. Given the rapid development of MinION-based sequencing, a <4 h time from positive blood culture to species identification of any organism, drug susceptibility prediction and phylogenetic placement is becoming increasingly tangible. Application to direct from blood/plasma sequencing would similarly reduce diagnostic time [35], and provide a crucial step towards point-of-care BSI diagnosis. However, in this investigation 3/9 MinION sequencing runs failed to generate sufficient data for analysis; suggesting optimization of methods to improve robustness is required. MinION sequencing may also prove to be prohibitively expensive, with uncertain potential surrounding the ability to multiplex samples and thus reduce costs.

We demonstrate that DNA of sufficient quantity and quality can be extracted from positive blood culture bottles to allow species identification and drug-resistance prediction using MiSeq- and MinION-based WGS. WGS offers the potential for an end-to-end diagnostic solution, replacing the multiple clinical workflows currently used to support species identification and drug susceptibility testing. Further investigations are required to assess the performance of WGS in parallel with routine clinical testing.

## Supplementary Data

626 Supplementary File 1
