# Supplementary material for: DNA extraction from primary liquid blood cultures for bloodstream infection diagnosis using whole genome sequencing
Source: J Med Microbiol. 2018 Jan 10;67(3):347–57. doi: 10.1099/jmm.0.000664 (PMC5882078; doi:10.1099/jmm.0.000664)
Supplement: Supplementary File 1 [file jmm-67-347-s001.pdf]

Supplementary material

Table S1: Quantitative PCR primers and probes

Organism	Target gene	Primer or Probe	Sequence (5’→3’)	Source
S. aureus	*nuc*	Forward	GTTGCTTAGTGTTAACTTTAGTTGTA	[1]
		Reverse	AATGTCGCAGGTTCTTTATGTAATTT	
		Probe	CY5-AAGTCTAAGTAGCTCAGCAAATGCA-BBQ	
E. coli	*uidA*	Forward	AAAACGGCAAGAAAAAGCAG	[2]
		Reverse	AYGCGTGGTTACAGTCTTGCG	[3]
		Probe	ROX-TGGACGATATCACCGTGGTGACGCA-BHQ2	[2]
Bacteria	16S rDNA	Forward	TCCTACGGGAGGCAGCAGT	[4]
		Reverse	GGACTACCAGGGTATCTAATCCTGTT	
		Probe	ROX-CGTATTACCGCGGCTGCTGGCAC-BHQ2	
Human	GAPDH	Forward	CCCCACACACATGCACTTACC	[5]
		Reverse	CCTAGTCCCAGGGCTTTGATT	
		Probe	FAM-AAAGAGCTAGGAAGGACAGGCAACTTGGC-TAMRA	
Human	β-actin	Forward	CCACACTGTGCCCATCTACG	[6]
		Reverse	AGGATCTTCATGAGGTAGTCAGTCAG	
		Probe	HEX-ATGCCCTCCCCCATGCCATCCTGCGT-BHQ1	

Table S2: DNA yield and purity for 6 samples extracted BiOstic (with and without SPRI clean-up) and 11 samples extracted with MolYsis kits. Mean, Standard Deviation (SD) and Interquartile Range (IQR) values are shown.

Measurement	Sample numbers	Extraction kit	*E. coli*			*S. aureus*		
			**Mean**	**SD**	**IQR**	**Mean**	**SD**	**IQR**
**Nanodrop (ng/µL)**	3x *E. coli*, 3x *S. aureus*	BiOstic	58.9	15.5	44.5 – 75.3	34.5	16.6	23.2 – 53.6
		BiOstic + SPRI	39.3	9.2	32.3 – 49.7	11.5	7.0	3.5 – 16.5
	5x *E. coli*, 6x *S. aureus*	MolYsis	1.6	0.7	1.2 – 1.7	3.0	1.4	2.3 – 3.3
**Nanodrop (260/280)**	3x *E. coli*, 3x *S. aureus*	BiOstic	1.4	0.7	0.9 – 2.1	0.9	1.1	0.2 – 2.2
		BiOstic + SPRI	2.0	0.1	1.9 – 2.2	1.9	0.5	1.4 – 2.2
	5x *E. coli*, 6x *S. aureus*	MolYsis	2.2	0.9	1.7 – 2.5	1.8	0.3	1.6 – 2.0
**Nanodrop (260/230)**	3x *E. coli*, 3x *S. aureus*	BiOstic	0.8	0.6	0.4 – 1.5	0.5	0.8	0.1 – 1.4
		BiOstic + SPRI	2.3	0.1	2.2 – 2.3	2.2	0.5	1.9 – 2.7
	5x *E. coli*, 6x *S. aureus*	MolYsis	0.1	0.0	0.0 – 0.1	0.1	0.0	0.0 – 0.1

Table S3: Assessment of differential centrifugation conditions. All DNA extracted using the BiOstic kit. Human copy number determined using β-actin target; qualitative assessment of bacterial DNA performed using 16S rDNA target.

Centrifugation speed (xg)	Centrifugation time (minutes)	Human qPCR copy number reduction (%)*		Bacterial cycle threshold†	
		Sample 1	Sample 2	Sample 1	Sample 2
**0**	**0**	N/A	N/A	18.11	16.02
**500**	**0.5**	48.24	88.00	17.46	15.92
	**1**	44.41	85.26	17.13	16.22
**1000**	**0.5**	53.33	90.96	17.37	16.92
	**1**	40.79	90.05	17.48	17.68
**2000**	**0.5**	76.80	83.51	17.73	16.91
	**1**	63.20	88.65	18.86	18.27
**3000**	**0.5**	53.60	77.32	18.12	17.32
	**1**	54.28	76.43	25.78	21.06

*β-actin qPCR

†16S rRNA qPCR

Table S4: Species identified by routine clinical diagnosis, taken as gold standard (Bruker microflex MALDI-TOF using pure culture isolates), and Illumina-based WGS with species prediction performed by Mykrobe (blood cultures positive for *Staphylococcus* species) or Kraken (blood cultures positive for Gram-negative organisms).

Routine species	WGS species	Number
**Blood cultures positive for Gram-positive species**		
Coagulase-negative staphylococci	Coagulase-negative staphylococci	36
Staphylococcus aureus	*Staphylococcus aureus*	14
Peptoniphilus harei	Coagulase-negative staphylococci	2
Coagulase-negative staphylococci	*Staphylococcus aureus*	1
Micrococcus sp.	Coagulase-negative staphylococci	1
**Blood cultures positive for Gram-negative organisms**		
Escherichia coli	*Escherichia coli*	19
Klebsiella pneumoniae	*Klebsiella pneumoniae*	3
Pseudomonas aeruginosa	*Pseudomonas aeruginosa*	3
Escherichia coli	*Escherichia coli + Klebsiella pneumoniae*	1
Klebsiella pneumoniae	*Klebsiella pneumoniae + Citrobacter koseri*	1
Klebsiella oxytoca	*Klebsiella oxytoca + Salmonella enterica*	1
Bacteroides fragilis	*Bacteroides fragilis*	1
Bacteriodes ovatus + Staphylococcus epidermidis	*Bacteroides* sp.	1
Escherichia coli + Streptococcus anginosus	*Escherichia coli*	1
Klebsiella pneumoniae + Acinetobacter lwoffii	*Klebsiella pneumoniae*	1
Citrobacter sp. + *Proteus miribilis*	*Citrobacter koseri + Proteus miribilis*	1
Escherichia coli + Citrobacter sp.	*Escherichia coli*	1
Escherichia coli + Enterococcus faecalis	*Escherichia coli + Enterococcus faecalis*	1
*Escherichia coli + Enterococcus faecalis + Pseudomonas aeruginosa**	*Escherichia coli + Enterococcus faecalis*	1
Escherichia coli + Klebsiella oxytoca†	*Escherichia coli*	1
Escherichia coli + Klebsiella pneumoniae	*Escherichia coli*	1
Klebsiella pneumoniae + Escherichia coli + Streptococcus oralis°	*Klebsiella pneumoniae*	1
Escherichia coli + Staphylococcus sp.	*Escherichia coli*	1

Klebsiella oxytoca + Pseudomonas aeruginosa	*Klebsiella oxytoca + Klebsiella pneumoniae*	1
Acinetobacter lwoffii	*Acinetobacter baumannii*	1
Aeromonas sp.	*Enterobacter aerogenes*	1
Brevibacillus sp.	Unclassified	1

**Pseudomonas aeruginosa* present in different (unsequenced) blood culture bottle from same patient

†*Klebsiella oxytoca* present in different (unsequenced) blood culture bottle from same patient

°*Escherichia coli* and *Streptococcus oralis* present in different (unseqeucned) blood culture bottle from same patient

Figure S1: Pre-extraction steps implemented and tested via qPCR, along with the finalised protocol. *Eppendorf 5810R centrifuge; †Thermo Scientific Heraeus Pico 17 centrifuge.

Human cell removal: pre-step a (n=2)

1. Centrifuge* 5 mL of culture:
   i. For 30s at 500 xg
   ii. For 1 minute at 500 xg
   iii. For 30s at 1000 xg
   iv. For 1 minute at 1000 xg
   v. For 30s at 2000 xg
   vi. For 1 minute at 2000 xg
   vii. For 30s at 3000 xg
   viii. For 1 minute at 3000 xg
2. Remove and retain supernatant, discard the pellet
3. Add equal volume ultrapure molecular grade water, vortex
4. Incubate for 5 minutes at room temperature
5. Centrifuge† at maximum speed (17,000 xg) for 2 minutes
6. Discard supernatant and re-suspend pellet in 450 µL Solution CB1
7. DNA extraction (BiOstic Bacteraemia)
8. 16S, human β-actin qPCR

Human cell removal: pre-step b (n=7)

1. Centrifuge* 5 mL of culture for 30s at 1000 xg
2. Remove and retain supernatant, discard the pellet
3. Add equal volume ultrapure molecular grade water, vortex
4. Incubate for 5 minutes at room temperature
5. Centrifuge† at maximum speed (17,000 xg)for 2 minutes
6. Discard supernatant and re-suspend pellet in 450 µL Solution CB1
7. DNA extraction (BiOstic Bacteraemia)
8. *S. aureus*, *E. coli,* human GAPDH qPCR

Human cell removal: pre-step c (n=6)

1. Filter 5 mL of culture through 5 µM membrane
2. Centrifuge* the flow through for 30s at 1000 xg
3. Remove and retain supernatant, discard the pellet
4. Add equal volume ultrapure molecular grade water, vortex
5. Incubate for 5 minutes at room temperature
6. Centrifuge† at maximum speed (17,000 xg) for 2 minutes
7. Discard supernatant and re-suspend pellet in 450 µL Solution CB1
8. DNA extraction (BiOstic Bacteraemia)
9. *S. aureus, E. coli,* human GAPDH qPCR

Finalised protocol

1. Centrifuge* 5 mL of culture for 1 minute at 2000 xg
2. Remove and retain supernatant, discard the pellet
3. Add equal volume ultrapure molecular grade water, vortex
4. Incubate for 5 minutes at room temperature
5. Centrifuge† at maximum speed (17,000 xg) for 2 minutes
6. Discard supernatant and re-suspend pellet in 1 mL nutrient broth with 10% glycerol for storage at -20°C
7. Defrost and centrifuge† stored samples at maximum speed (17,000 xg) for 3 minutes
8. Discard supernatant
9. Re-suspend pellet in 450 µL Solution CB1 and continue with BiOstic Bacteraemia extraction protocol following the manufacturer's instructions
10. Elute DNA in 100 µL Solution CB5
11. Purify DNA in 1.8x AMPure XP beads following the manufacturer's instructions
12. Elute DNA in 50 µL 1x TE buffer

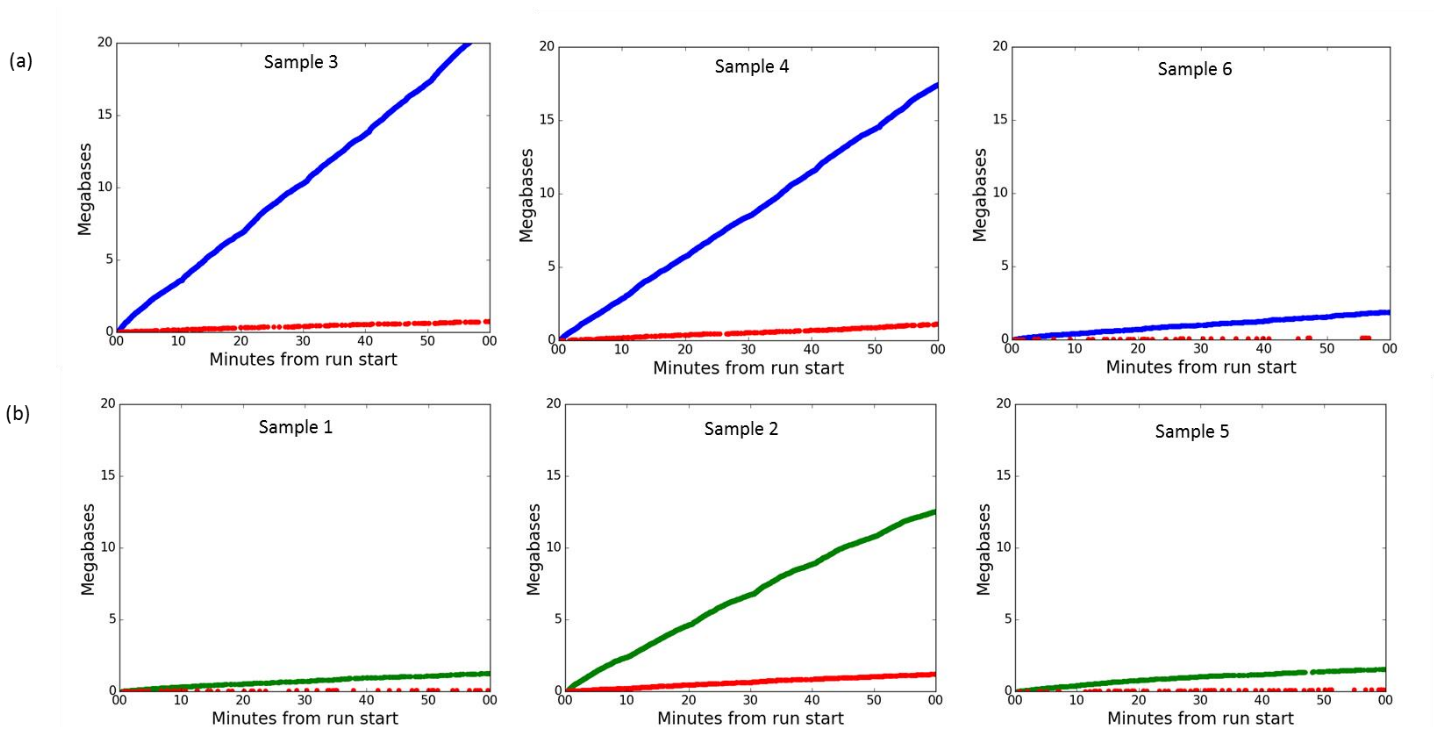

Figure S2: First hour of each MinION sequencing run. Reads mapped to Kraken in real-time; a) *S. aureus* positive samples, b) *E. coli* positive samples. Blue = *S. aureus*, green = *E. coli*, red = other bacterial DNA.

BIBLIOGRAPHY

1. **Kilic A, Muldrew KL, Tang YW, Basustaoglu AC**. Triplex real-time polymerase chain reaction assay for simultaneous detection of Staphylococcus aureus and coagulase-negative staphylococci and determination of methicillin resistance directly from positive blood culture bottles. *Diagn Microbiol Infect Dis* 2010;66(4):349-355.
2. **Bej AK, DiCesare JL, Haff L, Atlas RM**. Detection of Escherichia coli and Shigella spp. in water by using the polymerase chain reaction and gene probes for uid. *Applied and environmental microbiology* 1991;57(4):1013-1017.
3. **Pankhurst L, Macfarlane-Smith L, Buchanan J, Anson L, Davies K et al.** Can rapid integrated polymerase chain reaction-based diagnostics for gastrointestinal pathogens improve routine hospital infection control practice? A diagnostic study. *Health Technol Assess* 2014;18(53):1-167.
4. **Nadkarni MA, Martin FE, Jacques NA, Hunter N**. Determination of bacterial load by real-time PCR using a broad-range (universal) probe and primers set. *Microbiology (Reading, England)* 2002;148(Pt 1):257-266.
5. **Zhong XY, Laivuori H, Livingston JC, Ylikorkala O, Sibai BM et al.** Elevation of both maternal and fetal extracellular circulating deoxyribonucleic acid concentrations in the plasma of pregnant women with preeclampsia. *American journal of obstetrics and gynecology* 2001;184(3):414-419.
6. **Herrera LJ, Raja S, Gooding WE, El-Hefnawy T, Kelly L et al.** Quantitative analysis of circulating plasma DNA as a tumor marker in thoracic malignancies. *Clinical chemistry* 2005;51(1):113-118.
